# Investigating object representations during change detection in human extrastriate cortex

**DOI:** 10.1111/j.1460-9568.2010.07443.x

**Published:** 2010-11

**Authors:** D Samuel Schwarzkopf, Juha Silvanto, Sharon Gilaie-Dotan, Geraint Rees

**Affiliations:** 1 Institute of Cognitive Neuroscience, University College London17 Queen Square, London WC1N 3AR, UK; 2 Wellcome Trust Centre for Neuroimaging, University College London12 Queen Square, London WC1N 3BG, UK; 3 Brain Research Unit, Low Temperature Laboratory and Advanced Magnetic Imaging Centre, Aalto University, School of Science and TechnologyEspoo, Finland

**Keywords:** change detection, extrastriate cortex, object representation, repetition, transcranial magnetic stimulation

## Abstract

Detecting a change in a visual stimulus is particularly difficult when it is accompanied by a visual disruption such as a saccade or flicker. In order to say whether a stimulus has changed across such a disruption, some neural trace must persist. Here we investigated whether two different regions of the human extrastriate visual cortex contain neuronal populations encoding such a trace. Participants viewed a stimulus that included various objects and a short blank period (flicker) made it difficult to distinguish whether an object in the stimulus had changed or not. By applying transcranial magnetic stimulation (TMS) during the visual disruption we show that the lateral occipital (LO) cortex, but not the occipital face area, contains a sustained representation of a visual stimulus. TMS over LO improved the sensitivity and response bias for detecting changes by selectively reducing false alarms. We suggest that TMS enhanced the initial object representation and thus boosted neural events associated with object repetition. Our findings show that neuronal signals in the human LO cortex carry a sustained neural trace that is necessary for detecting the repetition of a stimulus.

## Introduction

Despite our subjective experience of a rich visual environment, our brain has only a limited capacity to process information about the world. When continuous viewing of a visual image is disrupted, as for example during a saccade or by flickering of the image, observers often find it particularly difficult to detect even considerable changes to the image. This ‘change blindness’ may involve a failure to encode and/or retrieve the memory of the original stimulus. Conversely, correct identification that no change has occurred must rely on a mnemonic trace of the visual image presented before the flicker.

Previous studies explored the extent to which visual disruptions can affect stimulus processing. When a change in a visual stimulus is not consciously perceived across such a disruption, the original stimulus can nonetheless exert an influence on the subsequent identification of a degraded target through priming effects seen behaviourally (Silverman & Mack, 2006; Yeh & Yang, 2009). This suggests that a representation of the original stimulus is maintained, but the neural locus for such a representation remains unclear. An undetected change can nevertheless evoke activity in functionally specialized regions of the ventral visual cortex, suggesting that these regions might be involved in such a persistent representation (Beck *et al.*, 2001). However, whether this is the case, plus the potential causal role of these regions, remains unclear. Previous studies using transcranial magnetic stimulation (TMS) implicate parietal and dorsolateral pre-frontal brain regions as having a causal role in change detection (Turatto *et al.*, 2004; Beck *et al.*, 2006; Tseng *et al.*, 2010). However, how far visual cortical regions carrying a representation of the stimuli are causally involved has not been explored.

Here we used TMS to test for the presence of a mnemonic trace of an object in the lateral occipital (LO) (Malach *et al.*, 1995) cortex and occipital face area (OFA) (Gauthier *et al.*, 2000), while participants attempted to discriminate whether an image of a familiar object had changed across a brief visual disruption. Previous studies show that TMS interacts with the initial state change of neuronal populations (for review, see Silvanto & Muggleton, 2008; Silvanto & Pascual-Leone, 2008; Silvanto *et al.*, 2008), including the state induced by visual short-term memory (VSTM) maintenance. When TMS is applied during a passive priming paradigm, it weakens the memory trace left by the prime (Campana *et al.*, 2002). In contrast, when TMS is applied during active VSTM maintenance, a behavioural effect reflecting the strengthening of the memory trace is found (Cattaneo *et al.*, 2009). These findings are consistent with functional magnetic resonance imaging (fMRI) evidence indicating the opposite neural effects of active and passive repetition priming paradigms (Soto *et al.*, 2007). Therefore, we reasoned with relation to our change detection task (which requires active maintenance of the first stimulus) that, if LO or OFA contained a neural trace of the original stimulus across the transient visual disruption, the application of TMS during the visual disruption should strengthen this memory trace and thus facilitate performance in the change detection task.

**Fig. 1:**
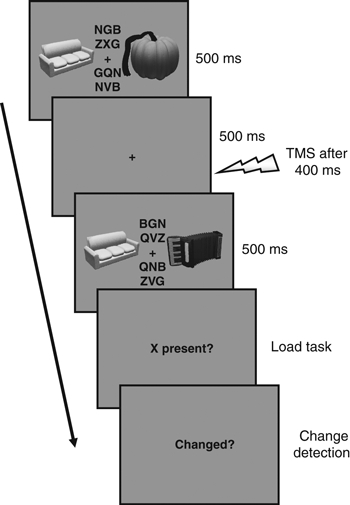
Schematic experimental ‘change’ trial. Participants viewed two successive intervals in each of which two images of household objects and four letter strings were presented around fixation as shown. After the second interval, participants were first asked to report whether the letter ‘X’ had been presented in either of the two intervals. Subsequently, they were required to indicate whether one of the two images of household objects had changed or not between intervals. In this example, an X was present (in the first interval) and the right-hand object underwent a change. In trials with TMS, a train of three pulses was administered at 20 Hz during the 100 ms preceding the second stimulus interval. Note that components of the stimuli are not to scale.

On each trial, participants fixated centrally and, as in a previous study (Beck *et al.*, 2001), performed a central letter detection task to increase the likelihood of change blindness. Simultaneously, images of two household objects were presented to the left and right of fixation in two successive 500 ms intervals separated by a 500 ms blank period (see Fig. 1). The number of objects and their positioning on the horizontal meridian were chosen to maximize the likelihood that they would be covered by receptive fields of neurons in the brain regions targeted by TMS. On some trials the identity of one object could change, whereas on others it remained unchanged. After each trial, participants were required to report whether one of the objects had changed during the trial. A TMS train was applied during the last 100 ms of the delay period between the original stimulus and the test stimulus (400 ms after the offset of the original stimulus) either to LO or OFA, or not at all.

## Materials and methods

### Participants

Eight healthy participants (five males and three females, all right-handed, mean age 27 years) gave written informed consent to take part in this experiment, which was approved by the local ethics committee (Institute of Neurology and National Hospital Joint Ethics Committee). All participants had normal or corrected-to-normal vision. Except for one of the authors (J.S.), all participants were naive to the purpose of the experiment. Inclusion of their data in the analysis was justified, however, because the results were qualitatively very similar with or without this participant. Moreover, the proximity of the TMS sites in this participant (1.43 cm Euclidean distance) made it very difficult for even an experienced participant to guess which site was targeted in a particular block. Data from one participant were excluded because of poor performance on the central letter detection task even in the No-TMS condition, at least two SDs below the group mean (88% correct) for all conditions.

### Stimuli

All stimuli were presented on an SVGA 17 inch monitor set at 1024 × 768 resolution and a refresh rate of 60 Hz using the Cogent package (http://www.vislab.ucl.ac.uk/Cogent/) for matlab (The Mathworks, Inc.). We used computer-generated images of household objects taken from the *Tarrlab* image database (http://tarrlab.cnbc.cmu.edu/stimuli.html). Images were converted to greyscale and normalized to their mean intensity value to minimize the influence of large luminance changes. The stimuli were presented on a uniform grey background. Each individual object image subtended 2.5° × 2.5° in visual angle. During each stimulus interval two images of household objects were presented, one to the left and one to the right of the central fixation cross at an eccentricity of 7.3°. In addition, two letter strings each comprising three letters were presented above and below fixation. The height of each letter string was approximately 1° so that all the letters combined subtended approximately 2.5° above and below fixation.

### Procedure

On each trial, participants viewed two stimulus intervals, each of which lasted 500 ms and which were separated by a 500 ms blank inter-stimulus interval (Fig. 1). On half of the trials, the same two images were presented in each interval. On the remaining trials a new image replaced either the left or right image. With a probability of 0.69 one of the letter strings in the trial contained the letter ‘X’.

After the second stimulus interval, participants first performed a letter-monitoring task – the instruction ‘X present?’ appeared in the centre of the screen and participants were required to indicate by means of two buttons on the computer keyboard whether they had seen the letter ‘X’ at any point in the trial. Subsequently, the instruction ‘Changed?’ appeared on the screen and participants were then asked to indicate using one of two buttons on the keyboard whether one of the images had changed between the two intervals or not. Participants responded with the index and middle finger of their dominant hand. The left and right arrow keys were used to indicate the presence and absence, respectively, of a change/target letter in both tasks. The order of the two tasks (letter task preceding change detection) was chosen to be comparable with the previous study using this procedure (Beck *et al.*, 2001) and to ensure adequate levels of difficulty in the change detection task.

The central letter-monitoring task was used to ensure that performance on the lateralized change detection task was not at ceiling for such a large change. In a brief training run immediately before the experiment, we adjusted the difficulty of the task on a per-participant basis between three levels of difficulty by varying the non-target letters in the strings to be more or less similar to the target ‘X’. In this pre-session, we aimed for participants to achieve an accuracy of approximately 85% correct on the central letter task, and performance on the change detection task away from ceiling and chance levels (70–80% correct). Rather than manipulating the signal strength of the stimuli, this procedure has been used previously (Beck *et al.*, 2001) in change detection experiments and has proved effective. Participants were also required to fixate for the entirety of a trial. We did not formally track eye movements but participants were monitored by the experimenter administering TMS. Moreover, because the changes of object images involved profound differences in low-level features between different objects, merely enforcing fixation by means of gaze-contingent eye tracking would not be sufficient to guarantee adequate levels of difficulty in the change detection task.

Altogether, each participant completed 384 experimental trials that were broken up into 12 blocks of 32 trials each. After every block, participants were informed about their performance on the central letter task. A different cortical site was targeted with TMS in different blocks: TMS could either be applied over LO (LO-TMS) or OFA (OFA-TMS), or not at all (No-TMS). The order of these conditions was pseudo-randomized using a permutation algorithm within and across participants. Each of the three TMS sites appeared exactly once in each triplet of blocks and the same site could never be stimulated two blocks in a row. None of the participants reported experiencing phosphenes as a consequence of TMS.

### Functional imaging

The fMRI was used to localize the sites for the TMS stimulation on a per-participant basis, due to their known variability in stereotactic location. A 1.5T Sonata system (Siemens) was used to acquire T1-weighted anatomical images and T2*-weighted echo planar images with BOLD (blood oxygenation level dependent) contrast. Each echo planar image was comprised of 32 2-mm axial slices with a 1 mm inter-slice gap covering the whole cerebrum with an in-plane resolution of 3 × 3 mm. The experiment was split into five runs, each consisting of 78 volumes. Volumes were acquired continuously with a TR (repetition time) of 2.88 s per volume.

The functional localizer scan used a one-back paradigm to focus attention. The three categories of visual stimuli were faces, objects and scrambled images of the objects. Each image subtended about 12° of visual angle and was presented for 360 ms with a 360 ms fixation interval between images. Participants were instructed to press a key whenever they detected an image repetition (one-back task) to ensure that they were alert and attentive. Stimuli were presented in blocks of 40 items from within a category, and a centrally presented red fixation cross was present throughout the experiment. There were two blocks of each stimulus condition per run. The order of categories was pseudo-randomized with the constraint that no two successive blocks could be of the same category.

All images were greyscale. To minimize retinotopic effects, we first generated phase-scrambled versions of the original images of all objects and faces, and then superimposed each image onto these scrambled images. Object stimuli created in this manner were further phase-scrambled to generate the scrambled category. This ensured that each category occupied the same area of visual space and that the spatial frequency and orientation content of objects and scrambled objects were identical. The localizer stimuli were also taken from the *TarrLab* image database.

Functional imaging data were analysed using spm5 (http://www.fil.ion.ucl.ac.uk/spm). After deleting the first four volumes of each run to allow for T1 equilibrium effects, the functional images were corrected for slice acquisition time, realigned to the first image using an affine transformation to correct for small head movements and echo planar image distortions unwarped using B0 field maps. The images were then smoothed with an 8 mm full-width half-maximum Gaussian filter and pre-whitened to remove temporal autocorrelation. The resulting images were entered into a participant-specific general linear model with three conditions of interest corresponding to the three categories of visual stimuli. Blocks were convolved with a canonical haemodynamic response function to generate regressors. In addition, the estimated motion parameters were entered as covariates of no interest to reduce structured noise due to residual head motion effects. Linear contrasts among the condition-specific regressors were used to identify the two TMS target sites within each participant’s right hemisphere: OFA by contrasting activation associated with face presentation to object presentation, and LO by contrasting objects with scrambled objects. The functional images were then registered to each participant’s individual structural scan using a 12 parameter affine transformation to identify two TMS target sites (OFA and LO) in the right hemisphere.

Each TMS target site was individually identified in each participant by selecting the peak activation for that category in the LO cortical region. The target sites corresponded well with previously reported maps of object- and face-sensitive regions (Hasson *et al.*, 2003; Pitcher *et al.*, 2009). The mean coordinates of the target sites in Montreal Neurological Institute (MNI) coordinates were: LO 44, −83, −7 mm; OFA: 45, −85, 3 mm. The important issue here is the within-participant separation between LO and OFA. The average Euclidean distance between the LO and OFA was 19.5 (±4.4) mm, providing a good separation of the two stimulation sites, which is also reflected in the functionally distinct effects of TMS reported in the present study (see below). Coordinates and Euclidean distances between sites for individual participants are shown in Supporting Table S1.

Although the stimuli used to localize the two TMS sites were not identical to those used in the TMS experiments, it is reasonable to use this procedure. The large localizer stimuli encompassed a significant portion of the visual field that contained the stimuli in the main experiment. Perhaps more importantly, the use of different localizer stimuli allows us to infer that the object-sensitive LO region identified by our localizer is indeed causally involved in our change detection task. Thus, rather than being specific to the particular stimuli used in our TMS experiment, this is tentative evidence that our results demonstrate a general property of LO.

### Transcranial magnetic stimulation

Rapid stimulator (Magstim, Wales) plus a 70 mm figure-of-eight coil were used for stimulation. A fixed TMS intensity (60% of maximum stimulator output) was used on the basis of a number of previous studies (e.g. Juan & Walsh, 2003; Muggleton *et al.*, 2003; Cattaneo *et al.*, 2010; Pitcher *et al.*, 2009). In the TMS conditions, a 20 Hz TMS train (i.e. with an inter-pulse interval of 50 ms) consisting of three pulses was applied on each trial during the inter-stimulus interval. The first pulse of the TMS train was applied at 400 ms after the offset of the first stimulus interval (and the last pulse at the onset of the second stimulus). The choice of this particular timing of TMS was to be consistent with previous state-dependent TMS experiments (e.g. Cattaneo *et al.*, 2010; Silvanto *et al.*, 2010) and to ensure that TMS was delivered close in time to the second stimulus but prior to the time when the second stimulus was encoded by LO neurons. The coil orientation was such that the coil handle was pointing upwards and parallel to the mid-line. Stimulation sites were localized using the brainsight TMS-magnetic resonance imaging co-registration software (Rogue Research, Montreal, QC, Canada), utilizing individual high-resolution structural magnetic resonance imaging scans for each participant. The right OFA and LO were localized by overlaying the structural magnetic resonance imaging scan with individual activation maps from the fMRI localizer task for the face and object analysis (as described in full above). The target area was identified by selecting the voxel exhibiting the peak activation in each functionally defined area and the coordinates were converted into BrainSight coordinate space using fsl software (http://www.fmrib.ox.ac.uk/fsl/). The coil locations were marked on each participant’s head using scalp marks and checked continuously during the experiment. Participants were instructed to inform the experimenter should they experience phosphenes; none of the participants did so.

## Results

In this experiment we sought to test whether stimulus representations in the object-sensitive LO and OFA cortex played a causal role in detecting whether a change occurred during repeated presentation of a visual object. Participants were asked to compare two stimulus intervals containing images of household objects and to report if one of the images had changed between intervals. We capitalized on the state dependency of TMS effects by applying TMS in the delay period between two stimulus intervals during which a trace of the visual memory of the first stimulus should be encoded and maintained in the visual cortex. We applied TMS over either LO or OFA, and measured how change detection performance differed from trials without TMS.

To maximize the occurrence of change blindness, participants performed a central letter detection task. One participant showed extremely poor performance on this central task and we therefore excluded this participant from any further analyses (see Materials and methods for details). For the remaining participants (*n* = 7), accuracy on the central task was high, but not at ceiling (No-TMS, 86.9 ± 1.2%; OFA-TMS, 88.1 ± 1%; LO-TMS, 89.7 ± 1.7%). Crucially, there was no significant difference in letter detection task accuracy comparing the three TMS conditions (i.e. LO-TMS, OFA-TMS or No-TMS; *F*_2,6_ = 2.39, *P* = 0.134). Moreover, there was no significant difference in the frequency with which the target letter appeared in different hemifields (*F*_2,12_ = 0.51, *P* = 0.615) or between the three TMS conditions (*F*_2,12_ = 0.09, *P* = 0.919) and no interaction between TMS condition and letter position (*F*_4,24_ = 0.68, *P* = 0.611). Thus, neither the use of a dual task nor the position of the target letters could have played any role in explaining our main results.

In addition to the central detection task, participants also performed a change detection task on the object stimuli that were presented concurrently. Without TMS, participants correctly reported a change in these stimuli on average following 77% of the trials. In other words, they exhibited change blindness on approximately one quarter of the trials. We calculated the participants’ sensitivity, d′, for detecting a stimulus change as well as the response bias for the three TMS conditions (No-TMS, OFA-TMS, LO-TMS).

**Fig. 2:**
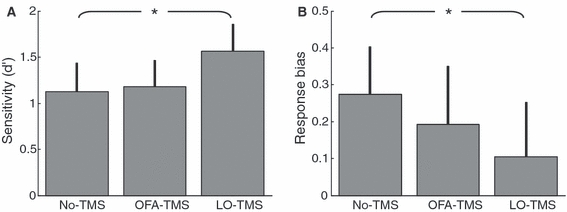
Sensitivity (A) and response bias (B) averaged across participants (*n* = 7) for the three TMS conditions (No-TMS, OFA-TMS, LO-TMS). Error bars denote 1 SE of the mean. Asterisks denote a significant difference between conditions (*P* < 0.02).

As Fig. 2A illustrates, sensitivity when TMS was applied over the OFA was similar to when no TMS was applied. In contrast, applying TMS over the LO caused a substantial increase in the sensitivity of participants for detecting change trials. This was supported by a significant main effect of the TMS condition in a one-way repeated-measures anova (*F*_2,6_ = 4.68, *P* = 0.031). This was due to the fact that, during LO-TMS, sensitivity was significantly greater than without TMS [*t*_6_ = 3.22, *P* = 0.018]. The effect was specific to stimulation of LO. Applying TMS over OFA resulted only in a moderate increase in sensitivity, which was not significantly different from trials without TMS [*t*_6_ = 0.37, *P* = 0.727]. TMS over LO also caused a shift in response bias (Fig. 2B) in the tendency of participants to report that a change had occurred (*F*_2,6_ = 5.53, *P* = 0.02). The response bias was significantly reduced for LO-TMS relative to the No-TMS condition [*t*_6_ = 4.0, *P* = 0.008] but not for OFA-TMS [*t*_6_ = 1.38, *P* = 0.218]. Although there was no significant difference between OFA-TMS and LO-TMS for either measure [sensitivity: *t*_6_ = −2.12, *P* = 0.079; response bias: *t*_6_ = 1.8, *P* = 0.123], the difference only between the LO-TMS and No-TMS conditions demonstrates that non-specific side-effects of TMS (e.g. scalp tapping, clicking noises, muscular effects) could not account for the results that we observed, as in that case we should also have found a significant difference between OFA-TMS relative to trials without TMS.

**Fig. 3:**
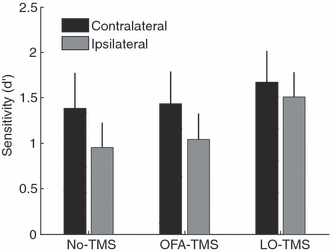
Sensitivity averaged across participants (*n* = 7) for detecting changes in the left (contralateral to TMS) or right (ipsilateral) hemifields for all three TMS conditions (No-TMS, OFA-TMS, LO-TMS). Error bars denote 1 SEM.

In all participants, the TMS sites were in the right hemisphere only. Because of the stimulus configuration, on half of the change trials the stimulus to the right of fixation changed (i.e. in the ipsilateral visual field with respect to the TMS site). Our images were small and presented at a relatively small eccentricity. Therefore, we expected that both images should fall within the large receptive fields of LO, which extend between 25–50% into the ipsilateral visual field (Dumoulin & Wandell, 2008). Nonetheless, for completeness we also analysed sensitivity separately for changes in the left and right visual field (Fig. 3). Again, there was a significant effect of TMS site (*F*_2,12_ = 4.06, *P* = 0.045). However, although the overall difference in sensitivity between hemifields approached significance (*F*_1,6_ = 5.34, *P* = 0.06), there was no interaction with TMS site (*F*_2,12_ = 1.24, *P* = 0.325), suggesting that TMS similarly affected change detection in the left and right visual field.

### Effect of transcranial magnetic stimulation as a function of trial type

**Fig. 4:**
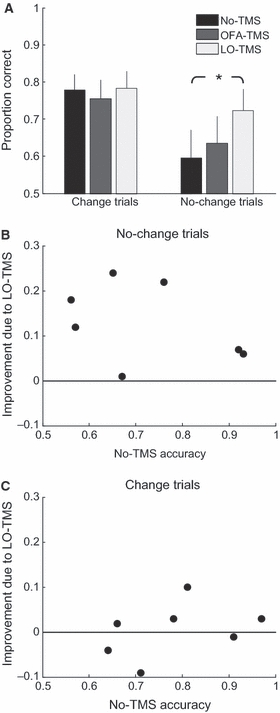
(A) Accuracy (proportion of correct trials) averaged across participants (*n* = 7) for the two types of trials (change trials, no-change trials) and the three TMS conditions (No-TMS, OFA-TMS, LO-TMS). Error bars denote 1 SEM. Asterisk denotes a significant difference between conditions (*P* < 0.01). (B and C) Improvement afforded by TMS over right LO (accuracy for LO-TMS minus No-TMS) plotted against the baseline performance without TMS. Data are shown separately for no-change (B) and change (C) trials.

The signal detection analysis presented above provides an overall measure of sensitivity and response bias in the change detection task. In order to investigate the impact of TMS in more detail, we carried out further analyses on participants’ accuracy for change and no-change trials separately (Fig. 4). Interestingly, applying TMS over LO caused a substantial increase of 13% in the accuracy on no-change trials (Fig. 4A), i.e. a reduction in the false alarm rate. In a two-way repeated-measures anova with factors TMS condition (No-TMS, OFA-TMS, LO-TMS) and stimulus change (change, no-change), there were no main effects of stimulus change (*F*_1,6_ = 2.34, *P* = 0.177) or TMS condition, although the latter approached significance (*F*_2,12_ = 3.54, *P* = 0.062); however, there was a significant interaction between these factors (*F*_2,12_ = 7.5, *P* = 0.008). This was due to the fact that, during LO-TMS, the accuracy for no-change trials was significantly greater than without TMS [*t*_6_ = 3.9, *P* = 0.008], but for change trials LO-TMS had no effect on performance [*t*_6_ = 0.25, *P* = 0.81].

In contrast, applying TMS over the OFA resulted in only a moderate increase in performance on no-change trials of 4% on average, which was not significantly different from trials without TMS [*t*_6_ = 1.01, *P* = 0.351]. Although there was also no significant difference between OFA-TMS and LO-TMS, this difference approached statistical significance [*t*_6_ = −2.4368, *P* = 0.0507]. Importantly, performance on no-change trials increased under LO-TMS, even for most of the participants who already showed high performance without TMS (Fig. 4B). Although at baseline for no-change trials many participants may have been guessing whether or not a change had occurred [one-tailed *t*-test against chance: *t*_6_ = 1.22, *P* = 0.134], this shows that even participants with above-chance performance had their detection of stimulus repetition improved by TMS over LO. Conversely, TMS did not cause even a subtle change in performance for change trials (Fig. 4C). Taken together, this analysis explains the findings for sensitivity (d′) and response bias reported above. Both signal detection measures are defined by the relationship between hit rate (correct change detections) and false alarms (errors on detecting stimulus repetitions). Because only the false alarm rate was modulated by TMS, the effects for sensitivity and the response bias that we observed were entirely driven by the false alarms, i.e. the accuracy of participants to correctly identify stimulus repetitions.

## Discussion

In this experiment we tested whether TMS could influence change detection across a visual disruption by targeting neuronal populations in human extrastriate cortex that are tuned to images of objects. We found that TMS applied over right LO cortex increased the sensitivity of participants in the task and also shifted their response criterion. Further analyses indicated that this effect was due to TMS specifically affecting no-change trials by reducing the false alarm rate. In contrast, TMS had no effect on change blindness; in other words it did not alter the ability of the participants to detect stimulus changes. Importantly, TMS had no effect on the performance on the central letter detection task that we employed to divide the participants’ attention and thus maintain adequate levels of change blindness. Therefore, our results cannot trivially be interpreted as TMS modulating the allocation of visual attention to between the centre of gaze and the peripherally presented object images. Further, because the significant effect of TMS relative to trials without TMS was specific for TMS over LO (and the difference between TMS over LO and OFA in identifying no-change trials approached significance), we are confident that our results are not accounted for by non-specific TMS side effects.

The pattern of results that we report here is consistent with a simple model of the neural events associated with the change or the repetition of a stimulus. The change detection task requires the participant to maintain an active representation, or neural trace, of the original stimulus. A subsequent presentation of the same stimulus interacts with this representation to signal that a repetition has occurred. The application of TMS to the brain area participating in this representation will modulate the effect of subsequent stimulus repetition. However, a different stimulus activates an entirely different neuronal representation, which is unaffected by the neural trace of the original stimulus. Therefore, TMS will only exert an influence on no-change trials. Note that TMS may interact with this signature of stimulus repetition either by directly affecting the response to the second stimulus or this may be a consequence of modulating the representation of the memory of the first stimulus during the delay period. In either case, the end result would be a behavioural effect for detecting a stimulus repetition.

This model, however, assumes that the neural representations of different objects are highly independent. If they are not, then for each two objects, their representations are very likely to overlap considerably. Thus, in our experimental setup, the representation of the target stimulus would not be completely different to the representation of the original stimulus, so that there would be an ensemble of neurons participating in both representations. This active ensemble of the representation of the original image would be affected by TMS in the same manner, whether it is a change trial or a no-change trial, making the difference between the two conditions indistinguishable. However, we do obtain a significantly distinguishable effect between the trial types when TMS is applied over LO, indicating that the neural representations of objects in LO are indeed highly independent and non-overlapping.

It is important to note that the precise nature of the representation of the object stimulus, whether an abstract encoding of the object or merely of the visual features from which it is composed, is not relevant for our interpretation. Single cell and optical imaging studies in macaque inferotemporal cortex and temporal cortex, which are considered the monkey homologue of human LO, seem to indicate that objects are represented in these regions by a pattern of responses over the population, and that the non-overlapping portion of different representations is large (e.g. Gross, 1992; Wang *et al.*, 1998). The fMRI adaptation studies (also termed repetition suppression) have also shown that viewing different objects causes release from fMRI adaptation in human LO cortex, indicating that the non-overlapping portion of different underlying representations is sufficiently large to elicit release from adaptation (Grill-Spector & Malach, 2001). It has recently been demonstrated that this is also true for different sub-exemplar representations of an unfamiliar object (inverted face) in LO (Gilaie-Dotan *et al.*, 2010). Here, in this study, we show the complementary effect, that the overlapping portion of different representations in LO is small and is differentially affected by TMS, indicating that the representations of different objects in LO are highly independent. Thus, the fact that TMS only had an effect on no-change trials provides further indications of the specificity of the TMS effects when a state-dependent paradigm is used.

Alternatively, it could be argued that the absence of a TMS effect for change trials was due to the fact that the baseline for the changed trials was higher than for no-change trials (cf. Fig. 4, No-TMS conditions). The performance level for change trials could be at the maximum that can be achieved under the load task, but there was room for improvement for no-change trials. The factor generally limiting performance may be the number of trials in which the observer failed to establish or maintain a representation of the original stimulus, because without this it is impossible to correctly identify changes as well as repetitions. It is possible that, for a proportion of no-change trials, the representation was present but too weak to generate a reliable repetition signal. By strengthening the representation, TMS elevated task performance to ceiling level. However, on change trials performance was already at ceiling even for these trials because a stimulus change is arguably more visually salient, and successful change detection can occur through comparison of local features (such as contrast edges and surfaces) even without a clear representation of the original object stimulus. However, the following reasons argue against this explanation. First, TMS led to an improvement in the detection of no-change trials for all participants, including those with high baseline performance. However, it did not consistently affect performance on change trials even for those with poor baseline performance (Fig. 4C). Second, as change and no-change trials differed both in terms of their physical stimuli and in their task demands, there is no reason to assume that the maximal performance that a participant could achieve was the same for both types of trial. Arguably, change trials were easier (because of differences in salience), which is supported by the fact that, for trials without TMS, the difference in performance between change and no-change trials approached significance.

We used the right OFA as a control TMS site, rather than stimulating a sham region like the vertex. Importantly, we defined these areas on a per-participant basis, thus ensuring that inter-participant variability in the location of individual areas did not affect our ability to distinguish between them in the group. As OFA and LO are both located in the occipital lobe, this ensured that the discomfort (from muscular stimulation, etc.) for both the site of interest and the control site were similar. Nevertheless, these brain regions are sufficiently far apart to be stimulated separately (Pitcher *et al.*, 2009). In the present study, as OFA-TMS had no impact on participants’ performance, we can be confident that the effect we observed that was associated with LO stimulation was indeed specific to the stimulation of that region and not due to a non-specific TMS effect. Importantly, we did not find a significant difference in the TMS effect for stimulus changes in the visual hemifield contralateral or ipsilateral to TMS. This could be taken as evidence that participants considered the two objects in each stimulus as one whole visual scene. However, this should have had no influence on the outcome of our TMS experiment, and we cannot make a specific interpretation for or against such an account from this null finding.

Previous TMS studies on change blindness have focused on the fronto-parietal network (Turatto *et al.*, 2004; Beck *et al.*, 2006; Tseng *et al.*, 2010). Beck *et al.* (2006) applied TMS over the right posterior parietal cortex and found a decrease in sensitivity in their signal detection analysis; this was due to a reduction in participants’ accuracy on change trials, implying that change detection was impaired. In that earlier study, the impact of TMS on the accuracy of no-change trials and on criteria in the signal detection analysis were not reported, precluding a direct comparison with the results of our study. Turatto *et al.* (2004) applied TMS over the right dorsolateral pre-frontal cortex and found a disruptive effect on change trials but not on no-change trials – the opposite pattern to that found here with LO TMS. These TMS results seem to fit well with fMRI evidence on change detection – the application of TMS over the pre-frontal cortex, a region associated with change detection in fMRI studies (Beck *et al.*, 2001), impairs change detection. In contrast, application of TMS over the extrastriate cortex (the activity of which does not discriminate between detected and undetected changes) (Beck *et al.*, 2001) has no impact on change detection, but rather appears to affect the strength of the memory trace. However, two issues complicate this conclusion. In the study of Turatto *et al.* (2004), the level of false alarms was very low (5% on average) and a ceiling effect could thus mask any TMS effect. Also, the TMS train in that study overlapped with the whole trial, and thus could have affected the encoding of the first stimulus, maintenance and encoding of the second stimulus. These issues were addressed by a very recent study by Tseng *et al.* (2010) in which TMS was applied over the right parietal cortex either during the encoding phase of the first stimulus or at the comparison phase. Reductions in change detection were found at both TMS time windows, indicating that the posterior parietal cortex is indeed critical for detecting change between two successive stimuli. Furthermore, in that earlier study no effects were found on false alarm rates, indicating that, unlike in our study, TMS over the parietal cortex does not shift participants’ response criterion.

The present result may appear to be inconsistent with findings that TMS can have the opposite, disruptive effect when applied in visual priming paradigms (Campana *et al.*, 2002, 2006; Cattaneo *et al.*, 2010). As both visual priming and VSTM rely on visual cortical activation traces outlasting target presentation, one might perhaps expect them to interact with TMS in the same manner. However, there is an important difference – VSTM and change blindness tasks encourage the active maintenance of visual information, whereas this is not the case in the visual priming paradigms previously used in conjunction with TMS. The importance of this difference was demonstrated in a recent fMRI study (Soto *et al.*, 2007) showing that the reappearance of a stimulus held in working memory enhances activity in occipital areas known to encode the prior occurrence of stimuli. In contrast, mere stimulus repetition elicits a suppressive response in the same regions (fMRI adaptation). Of course, state-dependent TMS and fMRI adaptation may be mediated by different neuronal mechanisms. However, the fact that TMS induces opposite effects in ‘active’ VSTM maintenance and ‘passive’ priming paradigms, although not easy to fit into the standard ‘virtual lesion’ conceptualization of TMS, is consistent with previous evidence (Soto *et al.*, 2007) of different neural states associated with stimuli held in working memory and passively observed targets. Our change detection task probably taps into the latter process because, in order to perform on this task, an observer needs to hold the original stimuli in memory. It could also be argued that TMS over LO facilitated performance in no-change trials by increasing the visual persistence of the first stimulus. This view would be consistent with a recent study in which TMS was found to bring attributes held in VSTM to visual awareness (Silvanto & Cattaneo, 2010). In the present study, such an effect would lead to an increase in the perceived duration of the first stimulus. Whether or not the behavioural facilitation occurred by a TMS/memory trace interaction in a manner that affected conscious visual perception, it is most parsimonious in the context of previous literature (Campana *et al.*, 2002; Cattaneo *et al.*, 2009, 2010) to argue that the present result was caused by an interaction between TMS and active VSTM maintenance.

As each stimulus interval in our paradigm contained a pair of object images presented to the left and right of fixation, we also conducted a separate analysis of change trials in which the change occurred in the visual hemifield contralateral and ipsilateral to the site of TMS. In accordance with previous TMS studies of LO and OFA, in all participants we only stimulated sites in the right hemisphere (Pitcher *et al.*, 2007, 2009). The overall difference in accuracy between hemifields approached significance, which could be seen as an indication that participants had a small bias towards detecting changes on the left. Crucially, however, there was no significant effect of TMS on detecting stimulus changes in either hemifield.

Taken together, our findings contribute to the growing body of evidence on the state dependency of TMS effects. The repetition of a stimulus is reflected in the responses of neurons that are tuned to that stimulus and neurons that sustain its percept. Our results provide evidence that LO plays a direct role in the signature of this repetition, and that this signature may be particularly susceptible to TMS.

## Abbreviations

fMRI: functional magnetic resonance imaging
LO: lateral occipital
OFA: occipital face area
TMS: transcranial magnetic stimulation
VSTM: visual short-term memory

## Supporting Information

Data S1. MNI coordinates of TMS sites (and the Euclidean distance between them in mm).

Please note: As a service to our authors and readers, this journal provides supporting information supplied by the authors. Such materials are peer-reviewed and may be re-organized for online delivery, but are not copy-edited or typeset by Wiley-Blackwell. Technical support issues arising from supporting information (other than missing files) should be addressed to the authors.
