# Supplementary material for: Investigating object representations during change detection in human extrastriate cortex
Source: Eur J Neurosci. 2010 Nov;32(10):1780–7. doi: 10.1111/j.1460-9568.2010.07443.x (PMC3003902; doi:10.1111/j.1460-9568.2010.07443.x)
Supplement: Supplementary file 1 [file ejn0032-1780-SD1.doc]

**Supplementary information**

Investigating object representations during change detection in human extrastriate cortex

D. Samuel Schwarzkopf, Juha Silvanto, Sharon Gilaie-Dotan and Geraint Rees

**Table S1**: MNI coordinates of TMS sites (and the Euclidean distance between them in mm)

|  |  | **OFA** |  |  |  | **LO** |  |  |  | **Distance** |
| --- | --- | --- | --- | --- | --- | --- | --- | --- | --- | --- |
|  |  | X | Y | Z |  | X | Y | Z |  |  |
| **Mean** |  | **44** | **-83** | **-7** |  | **45** | **-85** | **3** |  | **19.5** |
| *StD* |  | *14* | *12* | *7* |  | *10* | *10* | *12* |  | *4.4* |
|  |  |  |  |  |  |  |  |  |  |  |
| S1 |  | 58 | -81 | 4 |  | 63 | -69 | 27 |  | 26.9 |
| S2 |  | 46 | -91 | -12 |  | 37 | -102 | -8 |  | 14.3 |
| S3 |  | 52 | -77 | -3 |  | 51 | -86 | 11 |  | 17.0 |
| S4 |  | 41 | -79 | -10 |  | 41 | -95 | -9 |  | 15.5 |
| S5 |  | 19 | -104 | 1 |  | 36 | -88 | -1 |  | 23.4 |
| S6 |  | 57 | -64 | -9 |  | 53 | -76 | 1 |  | 16.8 |
| S7 |  | 53 | -78 | -10 |  | 40 | -81 | 8 |  | 21.9 |
| S8 |  | 27 | -92 | -14 |  | 41 | -82 | -4 |  | 20.1 |
